# Irradiation Damage Behavior and Mechanism of Pressureless-Sintered ZrC Ceramics

**DOI:** 10.3390/ma19102158

**Published:** 2026-05-21

**Authors:** Junping Ma, Haibo Wu, Huan Liu, Yitian Yang, Zehua Liu, Xishi Wu, Bingbing Pei, Jianshen Han, Canglong Wang, Zhengren Huang

**Affiliations:** 1 School of Material Science and Chemical Engineering, Ningbo University, Ningbo 315211, China; majunping@nimte.ac.cn; 2 State Key Laboratory of Advanced Marine Materials, Ningbo Institute of Materials Technology and Engineering, Chinese Academy of Sciences, Ningbo 315201, China; liuhuan@nimte.ac.cn (H.L.); yangyitian@nimte.ac.cn (Y.Y.); liuzehua@nimte.ac.cn (Z.L.); wuxishi@nimte.ac.cn (X.W.); peibingbing@nimte.ac.cn (B.P.); hanjianshen@nimte.ac.cn (J.H.); 3 Qianwan Institute of CNITECH, Qianwan New Area, Ningbo 315336, China; 4 Institute of Modern Physics, Chinese Academy of Sciences, Lanzhou 730000, China; clwang@impcas.ac.cn

**Keywords:** zirconium carbide, irradiation damage behavior, defects, thermal conductivity

## Abstract

Zirconium carbide (ZrC) is a leading candidate for advanced nuclear reactor components due to its ultra-high melting point, thermomechanical stability, and low neutron absorption. However, its irradiation damage behavior and mechanism remains underexplored. In this work, dense pressureless-sintered ZrC ceramics with low-neutron-absorption MoSi_2_ additives were irradiated with 500 keV He^2+^ ions at room temperature to peak damage levels of 0.30, 1.49, and 2.97 dpa. The changes in their microstructure, bonding states, and property were analyzed via TEM, GIXRD, Raman spectroscopy, nanoindentation, and TDTR. ZrC retained crystallinity regardless of high-density black-spot defects, while MoSi_2_ exhibited severe amorphization and swelling. Lattice expansion and partial Zr-C bond breakage with C-C bond formation were confirmed, with maximum hardening at 1.49 dpa and significant elastic modulus reduction at 2.97 dpa. Thermal conductivity decreased modestly and showed minimal dose dependence, indicating a saturation effect. These results elucidate defect evolution in pressureless-sintered ZrC-MoSi_2_ ceramics and support its application in high-irradiation nuclear environments.

## 1. Introduction

As part of the ongoing development of next-generation nuclear reactors under the Gen-IV program, certain designs are intended to operate in extreme service environments—specifically, at very high temperatures (1000–1200 °C under normal conditions and up to 1500 °C during off-normal or accident scenarios) and under high fast neutron fluxes [1,2]. Structural materials for these applications must therefore deliver exceptional high-temperature thermomechanical performance, combined with excellent chemical stability, corrosion resistance, and a low neutron absorption cross-section.

Zirconium carbide (ZrC), a transition metal carbide, exhibits a distinctive combination of desirable properties, including an ultra-high melting point (>3273 K), outstanding thermomechanical stability, excellent oxidation and corrosion resistance, oxygen-gettering ability, high thermal conductivity at elevated temperatures, and an exceptionally low neutron absorption cross-section [3,4]. These attributes position ZrC ceramics as promising candidates for advanced nuclear applications, such as inert fuel matrices in Generation IV gas-cooled reactors and structural components in fusion systems [5].

Several fabrication techniques have been developed for ZrC ceramics, including hot pressing (HP), hot isostatic pressing (HIP), spark plasma sintering (SPS), and pressureless sintering. Among these, pressureless sintering offers superior scalability and cost-effectiveness, particularly for producing large or geometrically complex components. However, due to strong covalent bonding and low self-diffusion coefficient of ZrC, sintering aids are typically required to promote densification. Conventional additives, such as those containing boron or rare-earth elements, often possess high neutron absorption cross-sections, which can compromise the radiation tolerance of the resulting ceramic. In contrast, MoSi_2_ has emerged as a promising sintering aid owing to its low neutron absorption cross-section, high melting point, excellent thermal conductivity, and outstanding corrosion resistance. In our previous work, we successfully fabricated highly dense ZrC ceramics via pressureless sintering using MoSi_2_ as a sintering additive, achieving exceptional thermal and mechanical performance [6]. This lays a solid foundation for further investigations into the irradiation behavior of ZrC ceramics.

In nuclear reactor environments, ZrC ceramics are inevitably subjected to neutron and ion irradiation, which lead to the generation, migration, and aggregation of various radiation-induced defects. These defects significantly influence the material’s microstructure and its resulting physical, mechanical, and chemical properties. Therefore, a comprehensive understanding of the irradiation damage behavior and the associated defect evolution mechanisms in ZrC ceramics is critical for assessing their structural reliability and long-term performance under irradiation.

Previous theoretical studies have contributed valuable insights into the irradiation response of ZrC. For instance, Jiang et al. employed ab initio molecular dynamics simulations to determine the average threshold displacement energies (E_d_) for C and Zr atoms in ZrC to be 34.5 eV and 41.5 eV, respectively, suggesting that carbon atoms are more readily displaced [7]. Their results indicate that Frenkel-type defects dominate the defect landscape. Similarly, Brutzel, using classical molecular dynamics, found that ZrC resists amorphization under irradiation, with only sparse formation of point defects such as carbon and zirconium interstitials [8]. Pellegrino et al. investigated the effects of 1.2 MeV Au ion irradiation on ZrC at room temperature, revealing the formation of Frenkel-like point defects at low fluences and extended dislocation-loop-like structures at higher fluences [9].

Additionally, Bao et al. observed the formation of black-dot defects (presumably small clusters of point defects) in ZrC_1−x_ ceramics following helium ion irradiation. The extent of lattice expansion exhibited a clear dependence on stoichiometry, following the sequence ZrC_0.85_ ≥ ZrC_1.0_ > ZrC_0.7_. Post-irradiation annealing led to a partial recovery of the lattice parameters, with the degree of recovery governed by both stoichiometry and annealing temperature [4]. Subsequent investigations revealed that annealing at 800 °C induced the formation of helium bubbles measuring 1–2 nm in diameter, whereas bubble coarsening and dislocation generation became pronounced at 1500 °C [10]. Collectively, these findings highlight the exceptional radiation tolerance of ZrC, as the material retains its crystallinity and resists amorphization across a broad spectrum of irradiation conditions. Nevertheless, the irradiation response of pressureless-sintered ZrC ceramics containing sintering additives remains insufficiently characterized and warrants further investigation.

In this work provides a systematic investigation of the irradiation response of pressureless-sintered ZrC ceramics containing MoSi_2_ as a low-neutron-absorption sintering additive—a material system that, to the best of our knowledge, has not been previously examined under ion irradiation conditions. Previous studies by our group have demonstrated that the addition of MoSi_2_ enhances the room-temperature mechanical properties of ZrC ceramics, but leads to a slight reduction in thermal properties [6]. Whereas prior research has largely centered on hot-pressed [11] or single-crystal ZrC [12], the present work addresses the knowledge gap concerning the irradiation tolerance of large-scale, pressureless-sintered ZrC ceramics, which are better suited for practical deployment in nuclear engineering applications.

This study employs a suite of advanced characterization techniques, including transmission electron microscopy (TEM), grazing-incidence X-ray diffraction (GIXRD), Raman spectroscopy, nanoindentation, and time-domain thermoreflectance (TDTR). Using these complementary methods, we elucidate the microstructural, bonding, mechanical, and thermal evolution of the ZrC matrix, together with the microstructural evolution of the MoSi_2_ secondary phase, following 500 keV He^2+^ irradiation at room temperature. The results reveal distinct damage behaviors between the ZrC matrix and the MoSi_2_ phase, clarify the nature of the induced defects and the mechanisms governing lattice expansion, and establish direct correlations between defect structures, irradiation-induced hardening, and thermal transport degradation. These insights not only advance the fundamental understanding of defect formation and stability in ZrC-based ceramics, but also offer practical guidance for the design of high-performance structural materials intended for next-generation nuclear energy systems.

## 2. Materials and Methods

The investigated ZrC ceramics were fabricated via pressureless sintering, as detailed in our previous work [6]. In this study, 5 vol% MoSi_2_ was added as a sintering aid. It promotes densification during the pressureless sintering process by forming a liquid phase. The bulk density was measured to be 6.64 g/cm^3^, corresponding to 98.8% of the theoretical value. The sintered bodies were cut into cuboid specimens of 5 mm × 4 mm × 2 mm. One face of each specimen was mirror-polished and subsequently subjected to ultrasonic cleaning and drying.

Ion irradiation experiments were conducted at room temperature using 500 keV He^2+^ ions with fluences of 1 × 10^16^, 5 × 10^16^, and 1 × 10^17^ ions/cm^2^. All irradiations were performed on the 320 kV multi-disciplinary highly charged ion research platform at the Institute of Modern Physics, Chinese Academy of Sciences.

**Figure 1:**
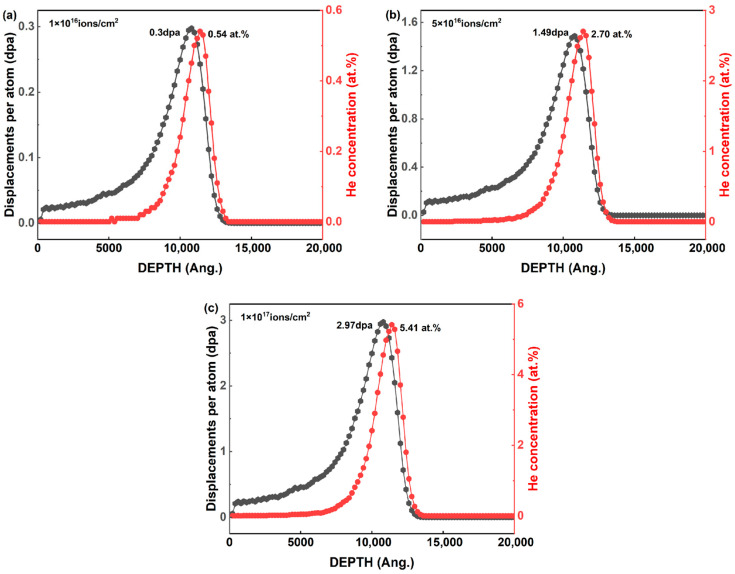
Depth profiles of displacement damage (dpa) and He concentration for ZrC irradiated with 500 keV He^2+^ ions at fluences of (**a**) 1 × 10^16^ ions/cm^2^, (**b**) 5 × 10^16^ ions/cm^2^, and (**c**) 1 × 10^17^ ions/cm^2^.

The displacement damage levels, expressed in displacements per atom (dpa), along with the ion concentration profiles, were computed using the “Full damage cascades with detailed calculation” option in the SRIM 2013 (Stopping and Range of Ions in Matter) simulation code. A ZrC density of 6.55 g/cm^3^ was assumed, with threshold displacement energies of 50 eV for Zr atoms and 21 eV for C atoms [13,14]. Notably, these effective E_d_ values were selected to align with standard benchmarks for carbides and Zr-based alloys, thereby compensating for in-cascade recombination. While ab initio calculations often yield lower values (e.g., ~41.5 eV for Zr), their direct use in SRIM typically overestimates defect production. Thus, the chosen parameters ensure both historical consistency and the reliability of the calculated dpa profiles. The dpa values and ion concentrations were determined according to Equations (1) and (2) [15]. For the three irradiation conditions, the peak damage levels were 0.30, 1.49, and 2.97 dpa, corresponding to peak He^2+^ concentrations of 0.54 at.%, 2.70 at.%, and 5.41 at.%, respectively, as shown in Figure 1. As can be observed, the asymmetric shape of this damage profile is a typical Bragg curve characteristic. High-energy He^2+^ ions initially lose energy mainly via electronic stopping with minimal atomic displacement, whereas nuclear stopping becomes dominant near the end of the ion range, creating the localized intense damage peak.

(1) $$dpa=vacancy(ions\times Å)\times \frac{10^{8}(\frac{Å}{cm})\times Fluence(\frac{ions}{{cm}^{2}})}{\rho (\frac{atoms}{{cm}^{3}})}$$

(2) $$at.\%ion=range(\frac{atoms/{cm}^{3}}{atoms/{cm}^{2}})\times \frac{Flunence(\frac{ions}{{cm}^{2}})}{\rho (\frac{atoms}{{cm}^{3}})}\times 100$$

TEM lamellae were prepared using a focused ion beam (FIB) system, integrated into a dual-beam scanning electron microscope (Helios 5 CX). The irradiation-induced microstructural damage was characterized by transmission electron microscopy (Talos F200X). The phase composition and lattice structure of ZrC before and after irradiation were analyzed by grazing-incidence X-ray diffraction (GIXRD, X′Pert^3^ Powder, Rigaku SmartLab, Tokyo, Japan) at a fixed incident angle of 2°. Raman spectra were collected at room temperature using a LabRAM HR Evolution spectrometer over the range 100–1800 cm^−1^ with a 532 nm excitation wavelength.

Nanoindentation tests were performed on both pristine and irradiated samples using an Agilent G200 nanoindenter equipped with a Berkovich diamond tip and operated in continuous stiffness measurement (CSM) mode, in which a small harmonic oscillation was superimposed onto the primary loading signal to determine the hardness and reduced elastic modulus as functions of indentation depth. A minimum of ten indentations was made for each sample to ensure statistical reliability. Thermal conductivity was measured using a nanosecond transient thermoreflectance (NanoTR) system at the Experimental Center for Engineering and Materials Science, University of Science and Technology of China. Due to the limited size of sample, each specimen was measured only once. Therefore, no statistical repeatability could be evaluated. The uncertainty of the TDTR measurement is estimated to be 5% according to the instrument specification and sensitivity analysis.

## 3. Results and Discussion

### 3.1. Microstructural Damage

**Figure 2:**
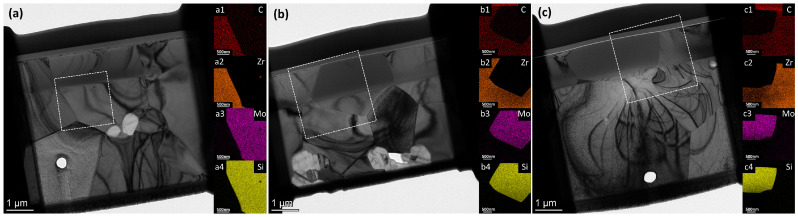
Microstructural and elemental evolution of the ZrC-MoSi_2_ ceramics under varying irradiation doses. Cross-sectional TEM images of the samples irradiated to (**a**) 0.30 dpa, (**b**) 1.49 dpa, and (**c**) 2.97 dpa. The corresponding EDS elemental mappings of C, Zr, Mo, and Si acquired from the white dashed boxes are shown in (**a1**–**a4**), (**b1**–**b4**), and (**c1**–**c4**), respectively.

**Figure 3:**
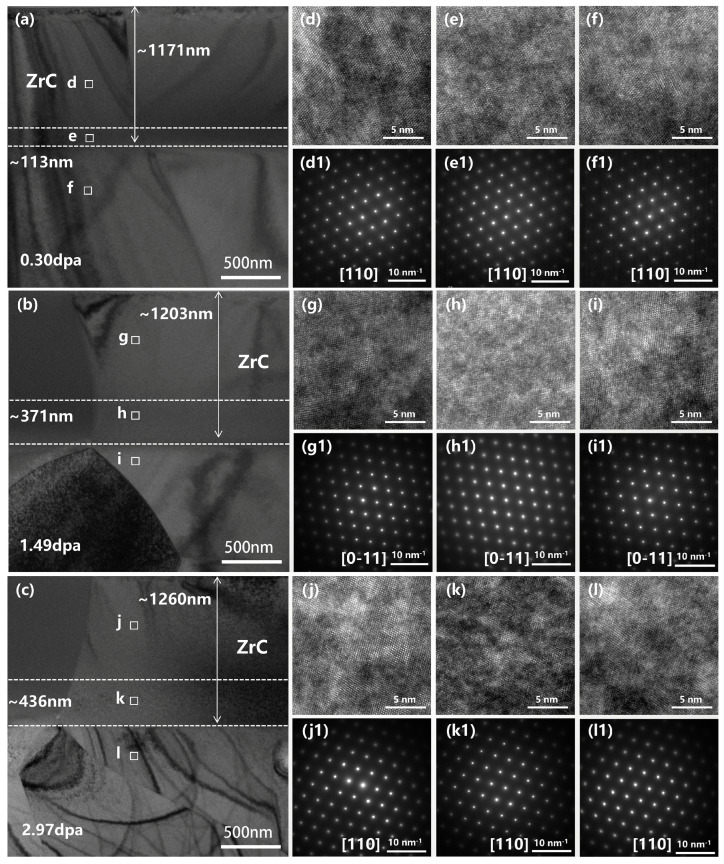
Main TEM micrograph of ZrC grains at (**a**) 0.30 dpa (**b**) 1.49 dpa (**c**) 2.97 dpa; (**d**,**g**,**j**), (**e**,**h**,**k**), and (**f**,**i**,**l**) correspond to high-resolution TEM images of the electronic stopping region, nuclear stopping region, and unirradiated region; below which (**d1**,**g1**,**j1**), (**e1**,**h1**,**k1**), and (**f1**,**i1**,**l1**) represent SAED patterns of their respective regions.

To investigate the microstructural and elemental evolution under varying irradiation doses, cross-sectional TEM observation and corresponding EDS mapping were performed on the ZrC-MoSi_2_ ceramics at 0.30, 1.49, and 2.97 dpa, as illustrated in Figure 2. Figure 3a–c present TEM micrographs of ZrC subjected to He^2+^ irradiation at fluences of 1 × 10^16^, 5 × 10^16^, and 1 × 10^17^ ions/cm^2^, corresponding to displacement damage levels of 0.30, 1.49, and 2.97 dpa, respectively. Experimentally, the geometric parameters of the damaged region were quantitatively measured from the cross-sectional TEM images using DigitalMicrograph software (Version 3.51.3720.0) based on diffraction contrast variations. The ‘total damage depth’ was defined as the perpendicular distance from the sample surface to the deepest boundary of visible defect contrast, while the ‘peak damage thickness’ was defined as the width of the intensely dark band corresponding to the maximum defect density. The associated damage depths and peak damage layer thicknesses for each dose are summarized in Table 1. The data reveal that both the total damage depth and the thickness of the peak damage layer increased with rising ion fluence. The damage in the peak region is primarily caused by collision cascades generated through nuclear stopping [16]. In this process, an energetic incident ion displaces a lattice atom in a direct knock-on event; the displaced atom then transfers its kinetic energy to neighboring atoms, initiating a chain of further displacements, known as a “displacement cascade” [17].

Noticeable changes in diffraction contrast were observed within the nuclear stopping region [18], indicating structural modifications induced by irradiation damage. Despite these effects, ZrC lattice structure remained largely intact. Even in the most severely damaged areas of the nuclear stopping zone, the diffraction spots exhibited only slight blurring, with no evidence of amorphization. These observations are consistent with earlier findings on irradiated ZrC [19]. For instance, Kim et al. reported an exceptionally high antisite defect formation energy for ZrC [20], which suggests a low propensity for chemical disorder-a condition often regarded as a prerequisite for amorphization. This inherent characteristic likely accounts for the remarkable resistance of ZrC to radiation-induced amorphization. Moreover, Zheng et al. provided a comprehensive analysis of this resistance, encompassing structural, thermodynamic, chemical, and kinetic perspectives [21]. Shi et al. suggested that the local accumulation of carbon vacancies promotes the recombination of irradiation point defects, thereby enhancing the irradiation tolerance of ZrC [22]. Additionally, diffraction spots located farther from the central transmitted beam displayed slight elongation and distortion, indicative of localized lattice strain. Such distortions are presumably attributable to various irradiation-induced defects.

**Figure 4:**
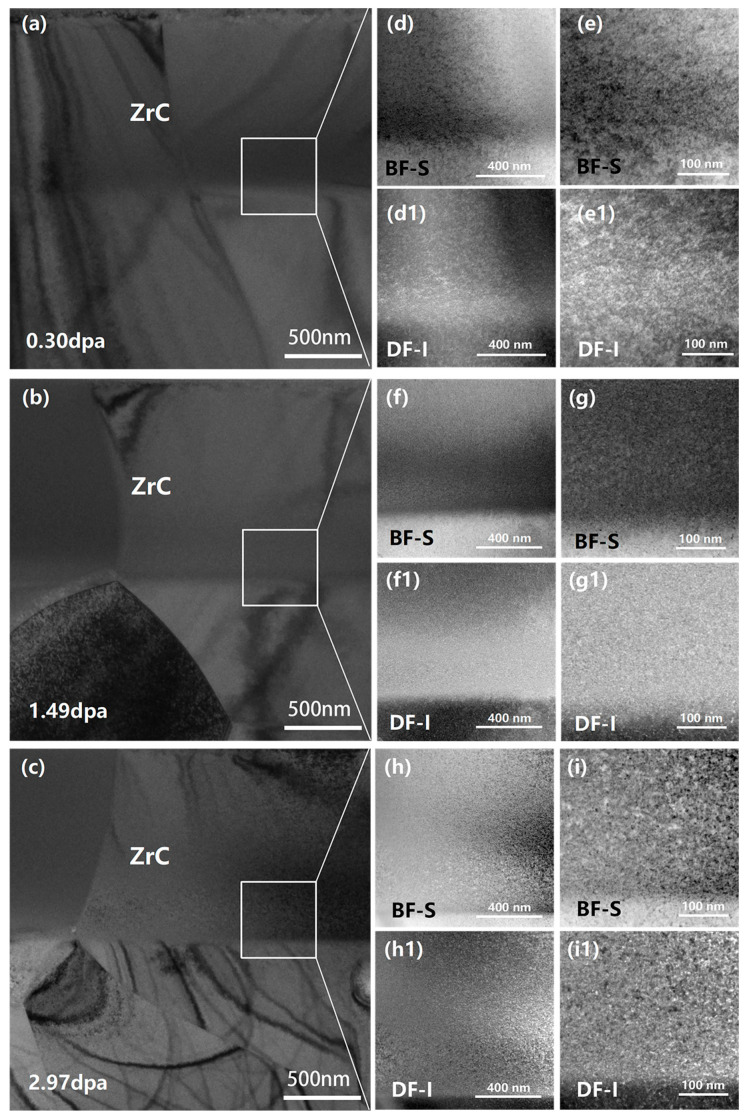
Bright-field (BF) and dark-field (DF) transmission electron microscopy (TEM) images of black-spot defects in the nuclear stopping region of ZrC grains at different irradiation doses: 0.30 (**a**), 1.49 (**b**), and 2.97 dpa (**c**). Panels (**d**,**f**,**h**) and (**d1**,**f1**,**h1**) show low-magnification BF and DF images, respectively; (**e**,**g**,**i**) and (**e1**,**g1**,**i1**) show the corresponding high-magnification BF and DF images.

TEM analysis confirmed that only black-spot defects were present in He^2+^-irradiated ZrC [23], as shown in Figure 4. Both bright-field (BF) and dark-field (DF) images revealed a progressive increase in defect size and density with rising irradiation dose. These black spots are most plausibly attributed to small interstitial clusters generated by ion irradiation, with dimensions falling below the resolution limit of the TEM. Notably, no helium bubbles were observed under any of the irradiation conditions examined, which is consistent with previous reports in the literature [24]. However, given that the practical TEM detection limit is on the order of 1–2 nm in diameter—depending on imaging conditions, local thickness, and contrast optimization—the presence of sub-nanometer helium-vacancy clusters cannot be completely excluded. Such ultra-fine defects may simply fall below the imaging resolution or manifest as nonspecific black-spot contrast.

**Figure 5:**
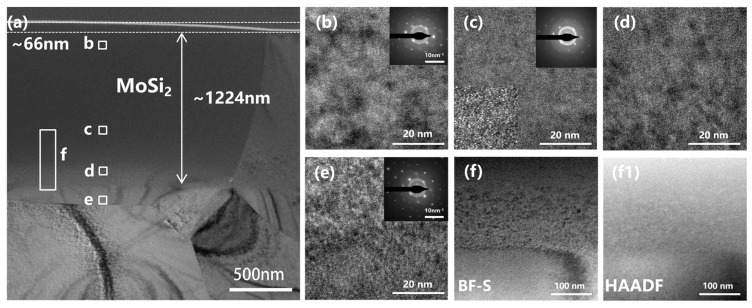
(**a**) TEM of the secondary phase (MoSi_2_) irradiated to 2.97 dpa; (**b**–**e**) High-resolution images with SAED patterns shown in the upper-right insets; (**f**,**f1**) Bright-field and HAADF images of the region “f”.

Additionally, TEM was employed to investigate the composition and irradiation-induced damage of the secondary phase. Figure 5 presents TEM micrographs of MoSi_2_ subjected to irradiation at 2.97 dpa. The measured damage depth in MoSi_2_ was approximately 1224 nm, which is slightly shallower than that observed in ZrC. As shown in Figure 5b,c, the severity of damage increased progressively with depth. Region b, situated near the surface, exhibited substantial irradiation damage, and the corresponding SAED pattern (Figure 5b) revealed the onset of amorphization. In Region c, although faint diffraction spots remained discernible, the microstructure had become nearly entirely amorphous, whereas previous studies have reported that MoSi_2_ can largely retain its crystalline structure under high-temperature irradiation conditions [25]. Region d, located at the terminal position of the nuclear stopping range, displayed a high density of black-spot defects, as clearly evident in Figure 5(f,f1).

Compared with Region e, the diffraction spots in Region b appeared considerably more diffuse, indicating that electronic stopping near the surface—though often considered a secondary effect—can nonetheless induce significant structural damage and therefore should not be overlooked. The pronounced amorphization also resulted in substantial volumetric swelling of the MoSi_2_ phase, as evidenced by TEM images showing that the irradiated region protruded approximately 66 nm above the ZrC surface. Notably, this difference in irradiation tolerance highlights that the ZrC matrix can successfully accommodate the severe localized swelling of the MoSi_2_ phase without inducing microcracking, which is crucial for the microstructural design of multiphase nuclear ceramics. Furthermore, the SAED patterns exhibited marked elongation and distortion of diffraction spots at increasing distances from the central transmitted beam, reflecting perturbations in the lattice structure. These distortions were particularly pronounced within the nuclear stopping region.

### 3.2. Lattice Expansion

**Figure 6:**
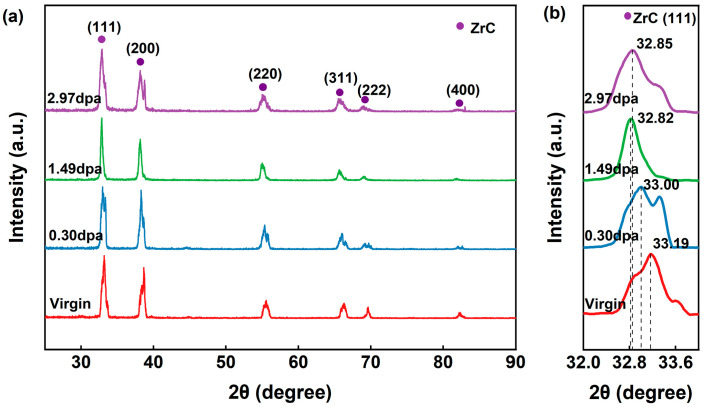
(**a**) GIXRD patterns of before and after irradiation; (**b**) enlarged view of the ZrC (111) diffraction peak.

GIXRD measurements were conducted at a grazing incidence angle of 2°, which corresponds to an approximate penetration depth of 0.9 μm, while this depth does not fully encompass the deepest tail of the overall damage region predicted by SRIM, it effectively captures the near-surface layer and the primary damage zone, allowing for a reliable assessment of the irradiation-induced structural evolution within this specific depth. Figure 6 displays the GIXRD patterns of ZrC ceramics before and after irradiation. Owing to the low MoSi_2_ content, its characteristic diffraction peaks were not detected. As shown in Figure 6a, the ZrC diffraction peaks exhibit distinct splitting and shoulder features, indicative of lattice defects. Notably, no evidence of amorphization was observed. The diffraction peaks of the unirradiated ZrC sample show asymmetric shoulder peaks and even slight peak splitting, which may be related to the material’s intrinsic carbon vacancies, residual stress introduced during the sintering process, and local lattice distortion. This indicates that the sample already had a certain degree of strain distribution before irradiation, while still retaining the face-centered cubic (FCC) crystal structure overall.

Figure 6b presents an enlarged view of the (111) diffraction peak of ZrC. Following irradiation, a systematic shift of the diffraction peaks toward lower 2θ angles is observed, indicative of irradiation-induced lattice expansion [26]. With increasing irradiation dose, the 2θ angles decrease progressively to 33.19°, 33.00°, 32.82°, and 32.85°, corresponding to fluences of 0, 0.30, 1.49, and 2.97 dpa, respectively. The calculated lattice expansion rates at these doses are 0.56%, 1.11%, and 1.00%, respectively. This lattice expansion is primarily attributed to the accumulation of black-spot defects, which disrupt the crystalline lattice. Additionally, Frenkel defects and helium interstitials are also expected to contribute [4]. Helium atoms can occupy tetrahedral and octahedral interstitial sites within the ZrC lattice, leading to further expansion. Previous studies have shown that Frenkel pairs constitute the most prevalent defect type in irradiated ZrC grains [7].

However, the peak shift observed at 2.97 dpa is slightly smaller than that at 1.49 dpa. This phenomenon likely arises because the internal lattice expansion exceeds that of the near-surface region, thereby exerting compressive stress on the outer layers and marginally reducing the apparent overall lattice expansion.

### 3.3. Bonding Evolution Behavior

**Figure 7:**
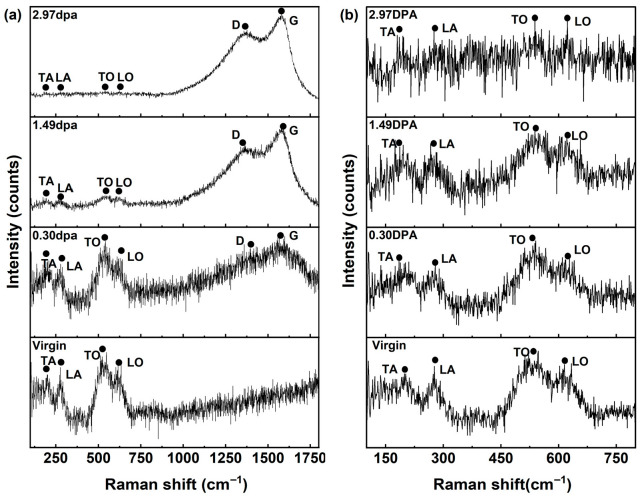
(**a**) Raman spectra of ZrC before and after irradiation; (**b**) Enlarged view of characteristic Raman peaks of ZrC.

Figure 7a and Figure 7b display the full-range Raman spectra and corresponding enlarged views of ZrC grains before and after irradiation, respectively. Prominent acoustic bands are located at approximately 198 cm^−1^ (TA) and 277 cm^−1^ (LA), while distinct optical bands appear at approximately 528 cm^−1^ (TO) and 615 cm^−1^ (LO). As shown in Figure 7b, the intensity of the characteristic ZrC peaks diminishes progressively with increasing irradiation dose, indicating cumulative structural degradation. This degradation impedes phonon propagation along the crystallographic axes [15], with the effect being most pronounced at 2.97 dpa. Figure 7a further reveals the emergence of new Raman bands at approximately 1364 cm^−1^ and 1581 cm^−1^, corresponding to the D-band and G-band, respectively. These bands are attributed to disordered carbon and graphitization [9], and are particularly evident at doses of 1.49 and 2.97 dpa. While trace surface carbon contamination deposited during the vacuum irradiation process may partially contribute to the Raman signal, the pronounced dose-dependent enhancement of the D and G bands indicates intrinsic structural evolution [27]. This observation suggests that He^2+^ irradiation of ZrC induces partial cleavage of Zr-C bonds, accompanied by the formation of C-C bonds. Consequently, such alterations are expected to impact the thermal and mechanical performance of ZrC, as will be examined in Section 3.4 and Section 3.5.

He^2+^ irradiation generated point defects in ZrC, which distort the local crystal structures, giving rise to the defect-induced Raman scattering described above. No significant shift in Raman peak positions was observed, likely due to the relaxation of internal stresses facilitated by grain boundaries in the polycrystalline material [9].

### 3.4. Irradiation Hardening

**Figure 8:**
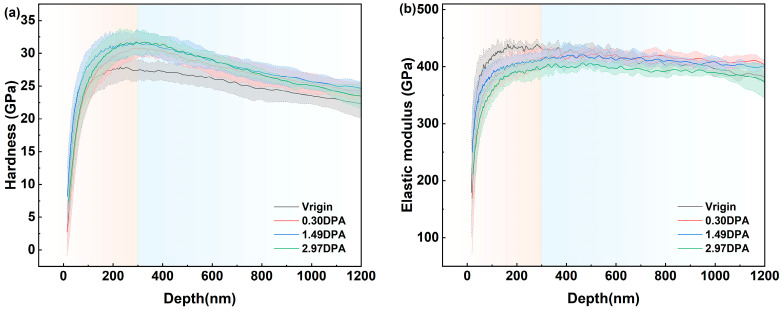
Variations of (**a**) nanohardness and (**b**) elastic modulus as a function of depth before.

To further evaluate the mechanical properties of ZrC ceramics before and after irradiation, nanoindentation tests were performed to obtain depth-dependent profiles of hardness and elastic modulus for all samples. To minimize grain orientation effects [28], at least 10 randomly selected indentations were carried out per sample, and the average values were used. The results are shown in Figure 8. Ion irradiation influences the mechanical behavior of ZrC through two competing mechanisms: (i) disruption of Zr-C chemical bonds, leading to hardness reduction, and (ii) generation of irradiation-induced defects—such as black-spot defects and defect clusters—that effectively hinder dislocation motion and slip, thus promoting irradiation hardening. Consequently, the observed changes in nano-hardness reflect the interplay between softening from bond breakage and hardening from defect pinning.

In Figure 8, the pink-shaded region corresponds to the indentation size effect (ISE) regime, where the data are considered unreliable. The blue-shaded region marks the irradiation-affected zone resulting from He^2+^ irradiation. As shown in Figure 8a, irradiation-induced hardening was observed in ZrC across all tested fluences. The most pronounced hardening occurred at 1.49 dpa, while the weakest was recorded at 2.97 dpa.

In the 300–1200 nm depth range, the average hardness values for the unirradiated, 0.30, 1.49, and 2.97 dpa samples were 25.07, 27.26, 27.78, and 27.44 GPa, representing increases of 8.7% (0.30 dpa), 10.8% (1.49 dpa), and 9.5% (2.97 dpa) relative to the pristine sample. These results are comparable to previous reports of a 10–15% hardness increase in ZrC_1.01_ irradiated to 1.5 dpa at 800 °C [29]. The hardening effect peaked at 1.49 dpa, suggesting a dose-dependent threshold between 0.30 and 2.97 dpa. This indicates that defect-induced dislocation pinning is the dominant mechanism. For instance, black-spot defects observed via TEM and defect clusters detected by Raman spectroscopy may both contribute to irradiation-induced hardening. Additionally, He^2+^ irradiation is known to introduce numerous interstitial-type defects, which can further enhance hardening.

As illustrated in Figure 8b, the elastic modulus exhibits distinct variations across irradiation doses. At 2.97 dpa, the modulus is lower than that of the unirradiated sample, while at 0.30 dpa and 1.49 dpa, it exceeds the unirradiated value beyond a depth of approximately 600 nm. The average moduli over the 300–1200 nm range are 408.25 GPa (unirradiated), 414.29 GPa (0.30 dpa), 410.50 GPa (1.49 dpa), and 393.47 GPa (2.97 dpa). Although irradiation hardening occurs at 2.97 dpa, the degradation of Zr-C bonds leads to a reduction in elastic modulus.

### 3.5. Thermal Conductivity

**Figure 9:**
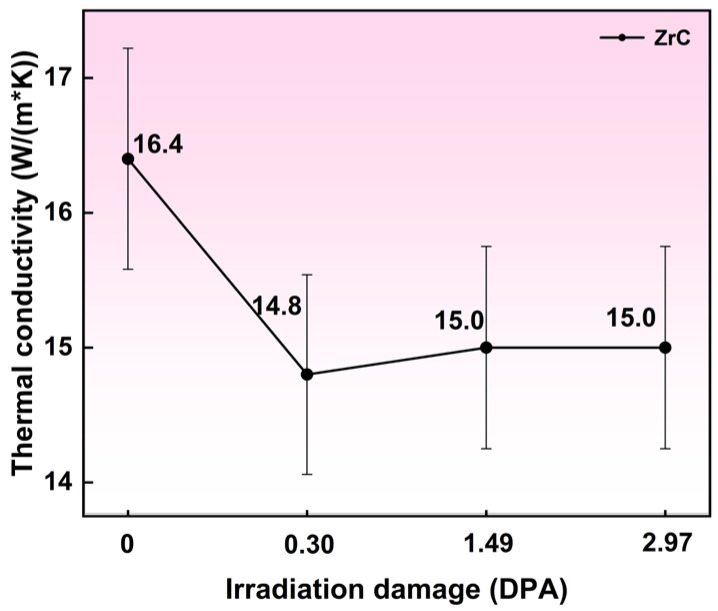
Variation in thermal conductivity before and after irradiation. Error bars represent the instrument uncertainty (±5%).

Compared with the conventional laser flash method, time-domain thermoreflectance (TDTR) provides a more effective means of characterizing the thermal properties of irradiated layers. Previous studies have indicated that for hot-pressed ZrC with atomic C/Zr ratios in the range of 0.6–0.9, the thermal conductivity typically falls between 8 and 11 W/(m·K). As shown in Figure 9, the room-temperature thermal conductivity of the unirradiated ZrC sample is 16.4 ± 0.82 W/(m·K), whereas that of the irradiated sample decreases slightly to approximately 15 ± 0.75 W/(m·K). Notably, a sharp reduction occurs at low doses and subsequently levels off beyond 0.30 dpa, indicating that ZrC retains excellent thermal conductivity even after ion irradiation—an observation consistent with previous reports [28]. Statistically, the initial ~8.5% decrease in thermal conductivity up to 0.30 dpa is significant as it exceeds the ±5% measurement uncertainty. However, the subsequent variations between 0.30 and 2.97 dpa remain well within the uncertainty limits.

It is well established that ion irradiation introduces a high concentration of vacancy defects, which constitute a major source of thermal conductivity degradation. Prior work has demonstrated that an increase in vacancy concentration leads to a corresponding decrease in lattice thermal conductivity [30]. In addition to vacancies, ion irradiation generates other defect types, including black-spot defects and disordered carbon and graphitization, as revealed by TEM and Raman spectroscopy. These defects enhance phonon scattering, shorten the phonon mean free path, and thereby impede thermal transport. Notably, the thermal conductivity values at 0.30, 1.49, and 2.97 dpa exhibit no significant variation, suggesting that a saturation or threshold level may be reached within this dose range.

The thermal conductivity saturation observed between 0.30 and 2.97 dpa originates from the combined effects of the phonon scattering limit and progressive defect evolution. At low doses, the proliferation of point defects drastically diminishes the phonon mean free path. Upon further irradiation, defect coalescence transforms the microstructure from dense, fine point defects to coarser, isolated clusters. Because these aggregated structures scatter phonons less efficiently than dispersed point defects, the accumulation of effective scattering centers saturates. As a result, the phonon mean free path reaches its theoretical minimum, stabilizing the thermal conductivity against additional irradiation.

## 4. Conclusions

ZrC ceramics were irradiated with 500 keV He^2+^ ions at room temperature. TEM observations revealed that both the damage depth and the thickness of the peak damage layer increased with irradiation dose. Irradiated ZrC exhibited a high density of black-spot defects, with no evidence of amorphization. In contrast, the MoSi_2_ secondary phase underwent pronounced amorphization accompanied by noticeable volumetric swelling. At all three irradiation doses, ZrC displayed varying degrees of lattice expansion, which is primarily attributed to the presence of black-spot defects. Helium interstitials further contributed to this expansion by occupying tetrahedral and octahedral interstitial sites within the lattice. Irradiation also induced cleavage of Zr-C bonds and the formation of C–C bonds, which may be associated with black-spot defects or exist as small carbon interstitial clusters. Nanoindentation measurements confirmed irradiation-induced hardening across all dose levels. Although the abundant defects enhanced hardness through dislocation pinning, the elastic modulus decreased markedly owing to extensive disruption of Zr-C bonds. Thermal conductivity declined under all irradiation conditions; however, the differences among doses were minimal, suggesting a possible saturation effect within this dose range.

## Figures and Tables

**Table 1:** Damage Characteristics under Different Displacement Damages.

Displacement Damage	0.30 dpa	1.49 dpa	2.97 dpa
Damage Depth (nm)	~1171	~1203	~1260
Peak Damage Thickness (nm)	~113	~371	~436

## Data Availability

The original contributions presented in this study are included in the article. Further inquiries can be directed to the corresponding authors.
